# Editorial: Reconstructive and aesthetic surgery of the face: new frontiers in research and clinical applications

**DOI:** 10.3389/fsurg.2026.1752335

**Published:** 2026-04-07

**Authors:** Mario Faenza, Roberto Grella, Giovanni Papa, Martin Iurilli, Marcello Molle

**Affiliations:** 1 Department of Medical and Surgical Sciences, University of Campania Luigi Vanvitelli, Naples, Italy; 2 Department of Plastic and Reconstructive Surgery, University of Trieste, Trieste, Italy

**Keywords:** aesthetic surgery, facial reconstructive surgery, minimally invasive techniques, non melanoma skin cancer, technological advancements, tissue regeneration

The face occupies a central place in human experience. It represents the primary medium of interpersonal communication and a fundamental component of individual identity, conveying emotions, intentions, and social cues. At the same time, it is one of the anatomical regions most frequently affected by cutaneous malignancies, often necessitating surgical excision followed by reconstruction (1). The dual functional and symbolic value of the face makes any therapeutic intervention particularly delicate, as surgical treatment inevitably extends beyond the mere removal of disease and may significantly influence a patient's appearance, self-perception, and quality of life (2).

For these reasons, considerable efforts have been devoted over the years to refining reconstructive strategies capable of achieving optimal oncologic control while preserving form and function. Techniques have evolved from secondary intention healing and skin grafting to increasingly sophisticated local and microsurgical flaps, allowing tailored solutions based on defect size, depth, and anatomical subunit.

A similar degree of attention has characterized the management of aesthetic concerns and age-related changes of the face, including tissue ptosis, volume depletion of facial fat compartments, blepharochalasis, and alterations in skin quality. Advances in surgical expertise and technological innovation have led to highly specialized and progressively less invasive procedures, such as endoscopic approaches in forehead lifting, as well as a growing integration of regenerative techniques and energy-based devices (3).

The aim of this Research Topic is therefore to provide a comprehensive overview of current standards and emerging perspectives in facial treatment, encompassing reconstructive and aesthetic surgery alongside regenerative medicine and minimally invasive or non-invasive therapies.

The collection brings together ten contributions addressing these diverse yet interconnected themes, offering updated insights and novel viewpoints in the management of facial pathologies and aesthetic subunits.

In the work of Chen et al., for example, the use of CO2 lasers was analyzed in comparison with standard surgical treatment for basal cell carcinoma: the use of this less invasive technique achieved comparable efficacy with reduced complication rates, while Brambullo et al. introduced a modification of the traditional forehead flap, known as the “dragonfly” technique. This variant enables the simultaneous reconstruction of both the internal and external nasal layers using a single vascular pedicle, thus allowing for total nasal restoration in a single stage.

Other works emphasize the importance of evaluating outcomes beyond purely technical success. Studies addressing minimally invasive cosmetic procedures (Alawadhi et al.) reveal the relevance of psychological assessment, underscoring that patient selection and informed consent must account for underlying anxiety or body image concerns. In parallel, investigations into blepharoplasty (Wang et al.) techniques and eyelid surgery document not only aesthetic improvement but also measurable gains in quality of life and reductions in appearance-related anxiety.

The work of Zhang et al. also evaluated eyelid cosmetic surgery, assessing transconjunctival blepharoplasty with orbital fat repositioning in 30 patients, noting not only an aesthetic improvement but also an improvement in quality of life.

When evaluating treatment for another facial area, the temporal fossa, a systematic review (Nasim et al.) supports the safety and effectiveness of autologous fat grafting for temporal hollowing, with high patient satisfaction and low complication rates.

Nasal surgery constitutes another major focus of the collection. Efforts to individualize clinically important differences in septoplasty outcomes highlight the need for patient-centered metrics in functional surgery (Haye et al.). At the same time, predictive models for complications in augmentation rhinoplasty (Ju et al.) and refined techniques for dorsal augmentation (Pincet et al.) illustrate the increasing role of risk stratification and technical standardization in aesthetic and reconstructive rhinoplasty.

The work of Gennai et al. analyzed the results of 22 years of application of the MIVEL (Minimal Incision Vertical Endoscopic Lift) technique, performed on 784 patients divided into three groups: MIVEL I (to intervene on the brow and periocular area in subjects between 21 and 35 years of age), II, and III (in subjects over 55 years of age with deeper facial sagging).

In conclusion, the contributions gathered within this Research Topic address several fundamental dimensions of facial surgery, spanning oncologic management, reconstructive strategies, aesthetic refinement, technical surgical considerations, and the psychological implications that inevitably accompany interventions in the cephalic region. Although often analyzed separately for methodological clarity, these domains are deeply interdependent in clinical practice. The treatment of facial conditions, whether driven by malignancy, trauma, congenital anomalies, or elective aesthetic indications, rarely allows for a compartmentalized approach, as each intervention carries functional, morphological, and emotional consequences.

Across all included works, a recurring message emerges: successful facial treatment requires a careful balance between oncologic radicality, reconstructive foresight, aesthetic harmony, and psychosocial awareness. The face is not merely an anatomical district but the core of individual identity and social interaction, and this complexity must guide every therapeutic decision.

Nevertheless, several challenges remain. The need for standardized and validated outcome measures that integrate functional, aesthetic, and psychological parameters is increasingly evident. Long-term prospective data are still limited for many minimally invasive and regenerative approaches. Moreover, the integration of predictive models and individualized risk assessment tools into routine practice represents an important future direction, particularly in rhinoplasty and reconstructive planning. Finally, strengthening multidisciplinary collaboration between surgeons, dermatologists, oncologists, and mental health professionals will be essential to ensure truly patient-centered care.
